# Improving circulating tumor cells enumeration and characterization to predict outcome in first line chemotherapy mCRPC patients

**DOI:** 10.18632/oncotarget.18025

**Published:** 2017-05-19

**Authors:** Luis León-Mateos, Helena Casas, Alicia Abalo, María Vieito, Manuel Abreu, Urbano Anido, Antonio Gómez-Tato, Rafael López, Miguel Abal, Laura Muinelo-Romay

**Affiliations:** ^1^ Axencia Galega de Coñecemento en Saúde (ACIS), SERGAS, Santiago de Compostela, Spain; ^2^ Liquid Biopsy Analysis Unit, Health Research Institute of Santiago (IDIS), CIBERONC, Complexo Hospitalario Universitario de Santiago de Compostela (SERGAS), Santiago de Compostela, Spain; ^3^ Translational Medical Oncology Group, Health Research Institute of Santiago (IDIS), CIBERONC, Complexo Hospitalario Universitario de Santiago de Compostela (SERGAS), Santiago de Compostela, Spain; ^4^ School of Mathematics, University of Santiago de Compostela (Campus Vida), Santiago de Compostela, Spain; ^5^ Roche-Chus Joint Unit for Precision Oncology, Health Research Institute of Santiago (IDIS), Complexo Hospitalario Universitario de Santiago de Compostela (SERGAS), Santiago de Compostela, Spain; ^6^ Research Unit for Molecular Therapy of Cancer, CNS Tumors, Vall d'Hebron University Hospital, Vall d'Hebron Institute of Oncology (VHIO), Barcelona, Spain

**Keywords:** prostate cancer, circulating tumour cells (CTCs), prognostic markers, taxanes resistance

## Abstract

**Introduction:**

There is a critical need of new surrogate markers for improving the therapeutic selection and monitoring of metastatic prostate cancer patients. Nowadays clinical management of these patients is been driven by biochemical and clinical parameters without enough accuracy to allow a real personalized medicine. The present study was conducted to go insight the molecular profile of circulating tumor cells (CTCs) isolated from advanced metastatic castration-resistant prostate cancer (mCRPC) with the aim of identifying prognostic marker with potential utility for therapy selection and monitoring.

**Materials and Methods:**

CTCs isolation was carried out in peripheral blood samples from 29 mCRPC patients that undergo systemic chemotherapy based on taxanes (docetaxel/cabazitaxel) and 19 healthy controls using in parallel CellSearch and an alternative EpCAM-based immunoisolation followed by RT-qPCR analysis to characterize the CTC population. A panel of 17 genes related with prostate biology, hormone regulation, stem properties, tumor aggressiveness and taxanes responsiveness was analysed to identify an expression signature characterizing the CTCs.

**Results:**

Patients with ≥ 5 CTCs/7.5ml of peripheral blood at baseline and during the treatment showed lower progression free survival (PFS) and overall survival (OS). Changes of CTCs levels during the treatment were also associated with the patient's outcome. These results confirmed previous data obtained using CellSearch in mCRPC. In addition, we found a CTC profile mainly characterized by the expression of relevant genes for the hormone dependent regulation of PCa such as *AR* and *CYP19* together with genes strongly implicated in PCa progression and resistance development such as *BIRC5*, *TUB1A*, *GDF15*, *RAB7* and *SPINK1*. Our gene-expression profiling also permitted the identification of valuable prognostic biomarkers. Thus, high levels of AR, CYP19 and GDF15 were associated with poor PFS rates while AR, GDF15 and BIRC5 were also found as reliable predictors of OS. Besides, a logistic model using KLK3 and BIRC5 showed a high specificity and sensitivity compared to CellSearch to discriminate patients with a more aggressive evolution.

**Conclusions:**

The molecular characterization of CTCs from advanced mCRPC patients provided with a panel of specific biomarkers, including genes related to taxanes resistance, with a promising applicability as “liquid biopsy” for the management of these patients.

## INTRODUCTION

Prostate cancer (PCa) is the most common diagnosed male malignancy in the Western world. For locally advanced and metastatic cancers androgen deprivation therapy is the standard of care. Despite its high response rates, most men eventually succumb to progressing disease, which has been termed castration-resistant prostate cancer (CRPC). The treatment landscape for patients with CRPC is progressing fast. In this context of advanced disease, systemic chemotherapy, new hormonal agents, immunotherapy and bone targeted therapies have shown an overall survival (OS) benefit [1]. Although the new therapeutic alternatives, chemotherapy remains an essential option to manage these patients [1].

The options for developing precision oncology in mCRPC patients are limited due to the few prognostic and predictive markers that are available for treatment selection and early evaluation of efficacy. Classically the key elements determining the prognosis and the decision of when to start or finish treatment in mCRPC patients are clinicopathological features, serum PSA and radiological evaluation [1]. Although their utility to manage the treatment, this approach is not enough to have an accurate evaluation of the disease prognosis and evolution. Moreover, currently we know that PCa is a dynamic disease, with different tumour clones emerging over time in response to different lines of therapy [2, 3]. Into this context, the clinical application of new surrogate markers will provide the opportunity for improving patient management and the therapeutic selection and monitoring.

Circulating tumor cells (CTCs) are tumour cells released into the blood from primary tumour or metastasis that emerged a decade ago as non-invasive alternative to interrogate the molecular profile of late stage tumours in comparison to invasive and sometimes inaccessible biopsy of metastatic disease. A considerable number of technologies have been developed to isolate, quantify and characterize CTCs in last years, but only CellSearch platform has been cleared by FDA for clinical use in metastatic breast, colorectal and prostate cancers [4–12]. Importantly, several studies have established the prognostic value of CTCs count for OS in patients with PCa. Thus, the presence of ≥ 5CTCs prior to the initiation of chemotherapy regimen was associated with lower OS [12]. Importantly, a decrease of CTCs count below five cells has been associated with higher OS similar to the benefit correlated to a substantial PSA decrease or radiographic response [13, 14]. Besides, changes in CTCs levels usually precede PSA fluctuation being their monitoring of even greater value when changes in PSA or bone disease are difficult to evaluate. Taking into account its prognostic value, CTCs enumeration by the CellSearch system has been investigated as a surrogate end-point for OS in different clinical trials [8]. Besides, CTC counts have been included in several phase I/II trials to monitor the efficacy of new treatments [15, 16].

In addition to the CTCs enumeration, the molecular characterization of CTCs will provide important insights into disease progression and might allow adaptation of therapeutic strategies, mainly in CRPC patients, since the optimal use of chemotherapy, enzyme inhibitors or AR antagonists require the application of precision molecular medicine. In fact, the presence of mutations, amplifications or splice variants of AR have been already assessed in CTCs with the aim to predict resistance to targeted treatments [17, 18]. In this sense, one drawback of the CTCs evaluation using the CellSearch technology is the lack of versatility to perform molecular characterization of isolated cells reducing its clinical impact.

In the present study we quantified and explored the molecular profile of CTC in mCRPC patients treated with taxane-based chemotherapy in order to identify CTCs markers with clinical value for the management of these patients. Our strategy combined an EpCAM-based inmunoisolation of CTCs and a RT-qPCR analysis of a panel of genes implicated in androgen-mediated signaling pathway, stem cell features, drug resistance or a more aggressive prostate tumor behavior. With this panel we evaluated critical steps and characteristics considered highly relevant for prostate CTCs biology [19, 20]. With this approach, previously validated in colorectal, endometrial and lung cancer [21–23], we identified a panel of biomarkers with demonstrated prognostic significance for progression free survival (PFS) and OS in mCRPC under taxane treatment.

## RESULTS

### Value of CTC enumeration in mCRPC patients under taxanes treatment

Enumeration of EpCAM positive CTCs from peripheral blood samples was performed using CellSearch system, as a well-accepted strategy for prostate carcinomas [5, 24]. CTCs were detected at baseline in 93.1% of patients, being the levels ≥ 5 CTCs/mL in 19 patients (65.5%). At 3rd and 6th treatment cycle the percentage of positive CTCs patients decreased to 55.1% and 31%, respectively, showing ≥ 5 CTCs/mL 13 (46%) patients at 3rd cycle and 6 (28.5%) patients at 6th cycle ≥ 5 CTCs/mL.

In addition we analyzed the correlation between clinicopathological features and the presence of ≥ 5 CTCs/7.5 mL. We found higher number of CTCs in patients diagnosed with locally advanced disease, nodal invasion, and also in patients that were responsive to hormonal therapy for less than 24 months. Interestingly, mean levels of serum AP and LDH were significantly higher when CTCs levels were ≥ 5 CTCs/7.5 mL (Supplementary Table 2).

**Figure 1:**
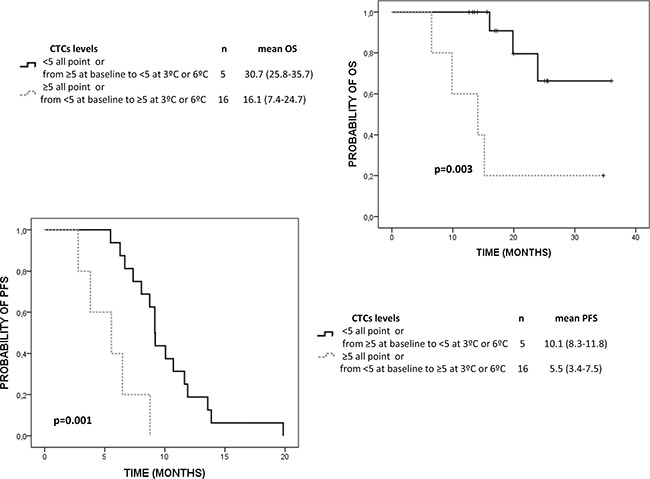
Kaplan Meier analysis for OS and PFS of CTCs levels changes within the treatment in mCRPC patients 5 CTCs/7.5 mL of blood was defined as the cut-off to separate the good and poor prognosis group.

Regarding the value of CTCs count as a prognostic and monitoring clinical tool, Kaplan-Meier analyses revealed a significant lower OS in patients with CTCs levels ≥ 5 CTCs/7.5 mL at all analysis points (Table 1). Importantly, CTCs levels was the unique variable with prognosis value, together with the presence of lymph none metastasis, to predict OS. Importantly, CTCs levels was the unique variable with prognosis value to predict OS together with the presence of lymph none metastasis. Higher CTCs levels were also associated with lower PFS but these differences were only significant at 6th cycle of treatment. In addition, CTCs changes within treatment also showed significant prognostic value to predict OS and PFS (Figure 1).

**Table 1:** Kaplan-Meier analysis for clinicopathological parameters and CTCs count

	Overall survival (OS)		Progression free survival (PFS)	
	mean (95% CI)	*p* value	mean (95% CI)	*p* value
**Performance status**				
PS0	31.2 (22.7–39.8)	0.12	8.1 (5.6–10.6)	0.92
PS1/PS2	22.9 (16.5–29.2)		7.6 (5.8–9.4)	
**Gleason Score**				
≤ 7	24.4 (17.8–30.6)	0.70	8.7 (6.3–11.1)	0.14
> 7	27.7 (17.8–37.5)		6.7 (4.8–8.7)	
**Lymph node metastases**				
no	**30.7 (23.4–38.1)**	0.05*	8.7 (6.7–10.7)	0.12
yes	**18.9 (11.4–26.5)**		6.3 (4.3–8.4)	
**N° of prior treatments regimens**				
≤ 2	22.03 (13.9–30.1)	0.17	6.5 (4.9–8.2)	0.09
> 2	28.4 (22.3–34.6)		9 (6.6–11.4)	
**Baseline PSA serum levels**				
≤ 122	25.7 (18.5–32.9)	0.68	7.6 (5.8–9.4)	0.83
> 122	24.4 (16.7–32.1)		7.9 (5.4–10.36)	
**Baseline LDH levels**				
≤ 320	24.3 (17.3–31.4)	0.67	7 (5.2–8.8)	0.71
> 320	22.9 (13.3–32.6)		6.8 (4.6–8.9)	
**Baseline PA levels**				
≤ 454	28.4 (22.3–34.6)	0.15	8 (6.5–9.5)	0.84
> 454	22.8 (14.4–31.4)		7.7 (5.1–10.9)	
**Baseline CTCs levels**				
< 5	**33.8 (30.2–37.5)**	0.007*	8.5 (6.7–10.2)	0.73
≥ 5	**20.5 (13.3–27.3)**		7.3 (5.3–9.4)	
**CTCs levels at 3rd cycle**				
< 5	**36.2 (29.6–42.8)**	0.001*	8.9 (7.4–10.4)	0.19
≥ 5	**17.3 (11.5–23.1)**		6.9 (4.4–9.5)	
**CTCs levels 6th cycle**				
< 5	**31.9 (26.9–36.9)**	0.003*	**10.3 (8.5–12.1)**	< 0.001*
≥ 5	**17.3 (9.8–25)**		**5.7 (3.9–7.4)**	

*p ≤ 0.05 according to Log-Rank test; HR: hazard ratio; CI: confidence interval.

We also evaluated the number of CTCs present before and after chemotherapy in patients that showed early progressive disease, in order to determine if an early increase in CTCs can anticipate tumor progression. We found that the 100% and 75% of patients showing respectively biochemical and radiological progression after 3 months had ≥ 5 CTCs/7.5 mL at 3rd cycle of treatment (*p* = 0.001 and *p* = 0.055 according to Ʃ^2^ test, respectively). Besides, the 100% and 80% of patients with biochemical and radiological progression after 3 months also maintain their levels over ≥ 5 CTCs/7.5 mL at 6^th^ cycle (*p <* 0.001 and *p* = 0.004 according to X^2^ test, respectively).

### CTCs molecular characterization in mCRPC

**Figure 2:**
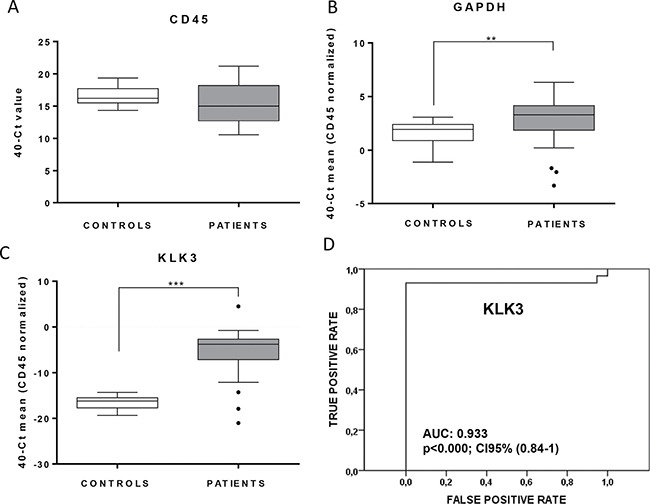
Validation of the CTCs isolation approach in mCRPC patients Box plots indicate median values in the group of control compared with the group of mCRPC patients for CD45 (**A**) GAPDH (**B**) and KLK3 (**C**) normalized to CD45. CD45, used as a marker of unspecific blood cells isolation showed no differences between both groups, while GAPDH and KLK3 demonstrated optimal accuracy for CTCs detection (**p < 0.01; ***p < 0.01 according to Mann-Whitney test). (**D**) ROC-curve showing the high sensitivity and specificity of *KLK3* to detect the presence of CTCs in our mCRPC cohort.

After CTCs immunoisolation using CELLection^TM^ Epithelial Enrich system we analysed the enriched fraction by q-RT-PCR. First, we evaluated the expression levels of *GAPDH* as a marker of cellularity, which includes both CTCs and unspecific blood cells, normalized to the background of *CD45* expression as specific marker for cells of hematopoietic origin [21]. As shown, *GAPDH* levels were significantly higher in the group of patients compared to controls (Figure 2A) indicating the presence of an extra population of cells isolated from the blood of CRPC patients. In addition, *CD45* did not present differences between both groups (Figure 2B), demonstrating that the unspecific background resulting from the process of immunoisolation was similar in the group of patients and controls. Importantly, when we compared the expression of *KLK3*, as an specific marker for prostate cells, no positive cases were found in the group of controls while 93,1% of patients were positive for *KLK3*, reinforcing the high specificity of our strategy for CTCs detection and analysis (Figure 2C and 2D). Globally, these results demonstrated the presence of CTCs in our cohort of CRPC patients.

Once demonstrated the efficiency of the CTCs isolation strategy, we explored the gene-expression profile of CTCs in samples from CRPC patients. We analyzed the expression levels of 15 genes in the whole set of patients and controls, and identified those genes with a significant expression in CTC from the group of patients compared to the background of unspecific isolation from the controls.

**Figure 3:**
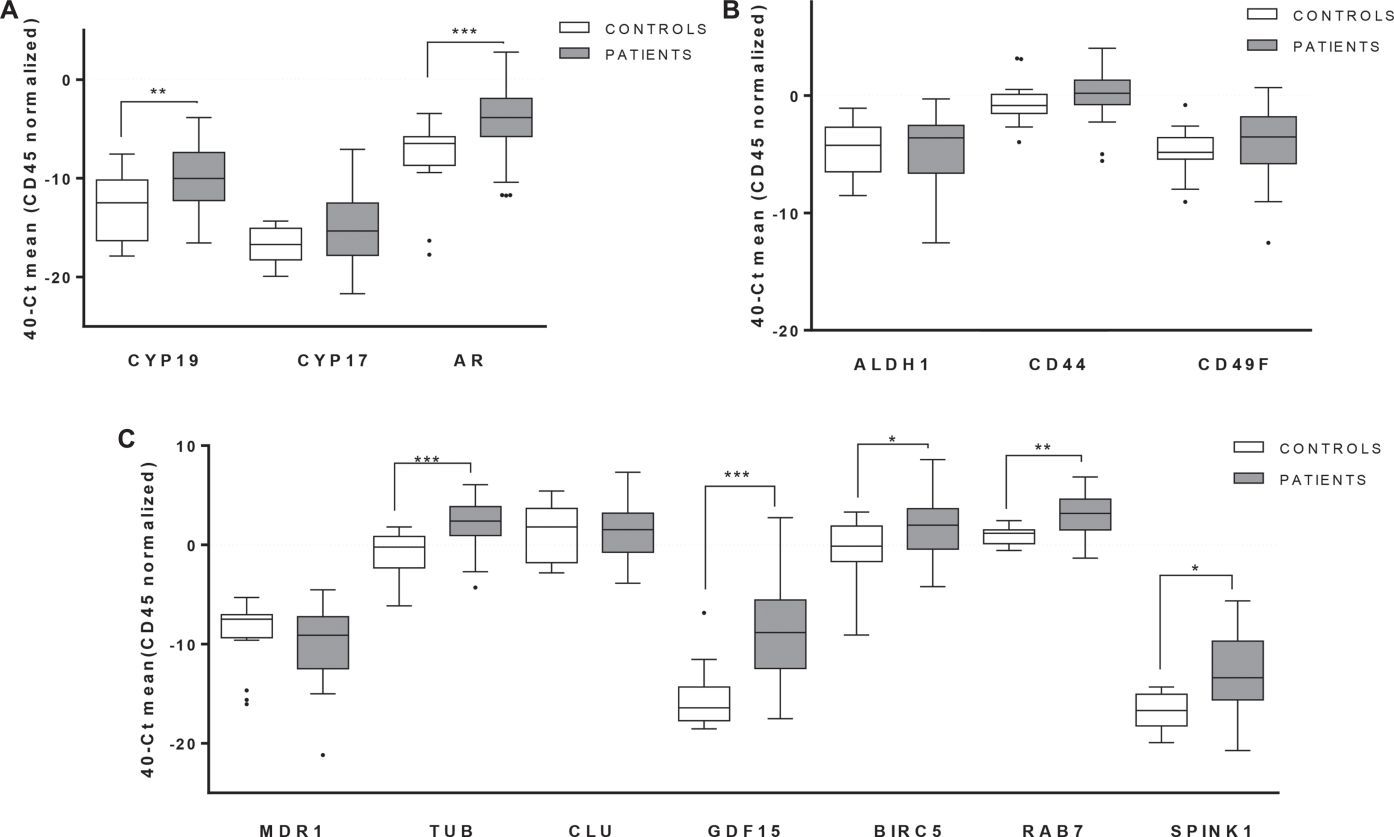
Gene expression profiling in CTCs from mCRPC patients Significant expression levels of genes involved in relevant signaling pathways for PCa biology: (**A**) hormone pathways (**B**) stem cell features and (**C**) associated with PCa progression and chemotherapy resistance. White boxes represent the gene expression levels in the group of healthy controls, grey boxes corresponding patients. (Mann-Whitney test, *p < 0.05; **p < 0.01; ***p < 0.001).

*CD133* and *MDR1* were expressed in less than 30% of patients, thus they were discarded for further analyses because they were not enough representative of the CTCs population. Among the remaining genes, we found significant higher expression levels in patient for *AR*, *CYP19*, *BIRC5*, *TUB1A*, *GDF15*, *RAB7* and *SPINK1* (Figure 3). All of them are considered to characterize the population of CTCs in our cohort of patients. This concern was reinforced after the analysis of Receiver Operating Characteristic (ROC) curves, showing all the validated genes high areas under the curves or AUROC, ranged from 0,70 (*BIRC5*) to 0,87 (*GDF15*) (Table 2).

**Table 2:** Diagnostic value to detect disseminated disease in mCRPC patients

	Receiver Operating Characteristic (ROC) curves		
GENES	AUC	*p*-value	CI 95%
*AR*	0.76	0.002	0.62–0.90
*CYP19*	0.74	0.006	0.59–0.80
*TUB1*	0.83	< 0.001	0.72–0.95
*GDF15*	0.87	< 0.001	0.76–0.98
*BIRC5*	0.70	0.024	0.54–0.86
*RAB7*	0.81	0.001	0.68–0.94
*SPINK1*	0.79	0.001	0.66–0.92

### Association between the CTCs profile and clinical parameters

We also analysed the possible association between standard clinical parameters and the levels of our CTC-markers and we found the results summarized in Supplementary Table 2. Overall, these results reflect the presence of greater levels of some CTC-markers in patients with poor clinical status before the treatment onset, in terms of PS, Gleason score and biochemical status.

### Prognostic value of the CTCs markers

In addition to the diagnostic value of our CTC-panel, we studied the prognostic impact of these markers to determine their real clinical interest for mCRPC patient's management. For that, we defined two groups of patients, those with low or high levels of each marker, using a cutoff defined as the 50, 60 or 70% percentile depending of each marker (Supplementary Table 4). We first investigated the prognostic potential of our CTC markers by Kaplan–Meier survival analyses for PFS and OS.

**Figure 4:**
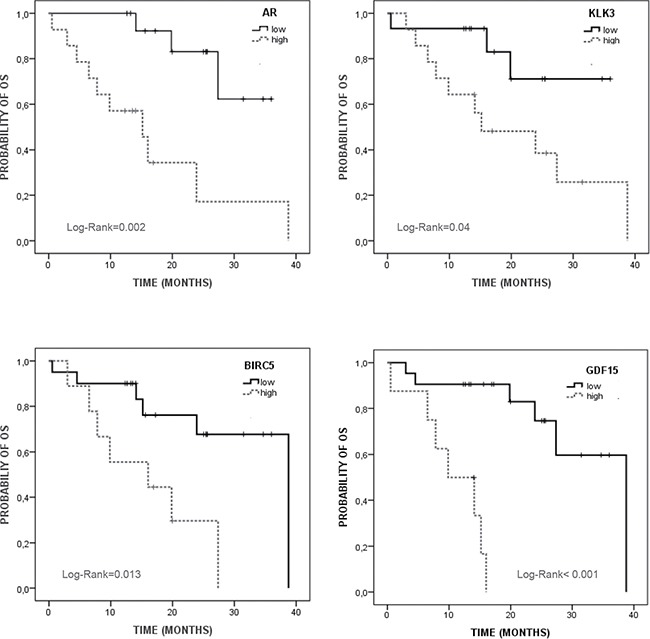
Kaplan Meier analysis for overall survival (OS) of validated CTC markers in mCRPC patients Low/high expression were defined based on the 50% (KLK3 and AR) and 70% (BIRC5 and GDF15) percentile (Supplementary Table 2).

As Table 3 shows, high levels of *KLK3, AR, CYP19* and *GDF15* were statistically associated with shorter PFS rates. For OS we found that patients with high levels of *KLK3, AR, GDF15* and *BIRC5* presented poorer survival rates than those with low levels (Figure 4). Thus, patients into the group of bad prognosis according to AR-CTC levels presented 16.6 months of OS while the good prognosis group reached a mean OS of 31 months (Table 3).

**Table 3:** Kaplan-Meier analysis for CTCs markers

	Overall survival (OS)		Progression free survival (PFS)	
	mean (95% CI)	*p* value	mean (95% CI)	*p* value
**KLK3**				
low	**29.65 (23.4–35.9)**	0.04*	**9.4 (7.2–10.6)**	0.012*
high	**20.4 (12.8–28)**		**5.9(4.4–7.4)**	
**AR**				
low	**31 (26.3–35.7)**	0.002*	**9.3( 7.2–11.5)**	0.002*
high	**16.6 (8.8–24.4)**		**6 (4.3–7.7)**	
**CYP19**				
low	26.6 (21.3–31.95)	0.12	**8.9 (7.2–10.6)**	0.015*
high	20.6 (9–32.2)		**5.2 (3–7.3)**	
**TUB1**				
low	21.5 (16–27)	0.18	8.5 (6.7–10.2)	0.09
high	30 (17.8–31.2)		5.7 (3.2–8.2)	
**GDF15**				
low	**31.1 (24.9–37.2)**	<0.001*	**8.6 (6.7–10.3)**	0.043*
high	**10.6 (6.8–14.5)**		**5.6 (3.5–7.7)**	
**BIRC5**				
low	**30.5 (23.7–37.3)**	0.013*	7.7 (6.2–9.3)	0.94
high	**15.8 (9.4–22.2)**		7.7 (4.2–11.2)	
**RAB7**				
low	19.9 (14.2–25.7)	0.11	8.5 (6.1–11)	0.22
high	30 (21.8–38.3)		6.9 (5.3–8.6)	
**SPINK1**				
low	23.1 (17.3–28.9)	0.58	8.5 (6.7–10.3)	0.12
high	28.4 (17.3–39.5)		6 (3.7–8.29)	

*p ≤ 0.05 according to Log-Rank test; HR: hazard ratio; CI: confidence interval.

Univariate Cox regression analysis confirmed the prognosis value of *KLK3, AR, CYP19* and *GDF15* to predict PFS while only *AR*, *GDF15* and *BIRC5* were confirmed as good predictors for OS. As Table 4 shows, patients with high *GDF15* levels presented a 2.5 and 15.7-fold increased risk of progression and death compared to patients with low *GDF15* levels. It is really important to remark that only these CTC-markers, among all the other clinical factors including in the study, were validated as prognostic markers after the univariate analyses. In fact, *KLK3* (PSA)-CTC associated levels were found as a good prognostic marker for both PFS and OS while serum PSA levels at baseline did not show any value to predict the response to the chemotherapy and the patient evolution (Table 4).

**Table 4:** Univariate Cox regression analysis for clinic-pathological parameters and CTCs markers

	Progression free survival (PFS)		Overall survival (OS)	
	HR (95% CI)	*p* value	HR (95% CI)	*p* value
**Performance status** (PS0 vs. PS1/PS2)	1 (0.43–2.4)	0.92	4.3 (0.55–34.2)	0.16
**Gleason Score** (≤ 7 vs. > 7)	1.8 (0.8–4.3)	0.15	0.78 (0.21–2.8)	0.7
**Lymph node metastases** (no vs. yes)	1.8 (0.83–3.89)	0.13	3.08 (0.91–10.3)	0.06
**N° of prior treatments regimens** (≤ 2 vs > 2)	0.5 (0.2–1.15)	0.10	0.44 (0.13–1.5)	0.18
**Baseline PSA serum levels** (≤ 122 vs > 122)	0.92 (0.42–1.9)	0.83	1.26 (0.4–4)	0.68
**Baseline LDH levels** (≤ 320 vs > 320)	0.85 (0.37–1.9)	0.7	1.3 (0.39–4.2)	0.67
**Baseline FA levels** (≤ 454 vs > 454)	0.92 (0.42–2)	0.84	2.4 (0.69–8.6)	0.16
**KLK3** (low vs high)	**2.7 (1.2–6.1)**	0.016*	3.52 (0.94–13)	0.06
**AR** (low vs high)	**2.5 (1.1–5.58)**	0.027*	**6.7 (1.7–25.6)**	0.005*
**CYP19** (low vs high)	**2.7 (1.16–6.24)**	0.020*	2.42 (0.75–7.76)	0.13
**TUB1** (low vs high)	2 (0.87–4.8)	0.09	0.36 (0.08–1.7)	0.2
**GDF15** (low vs high)	**2.4 (1–5.8)**	0.05*	**15.7 (3.1–79.7)**	0.001*
**BIRC5** (low vs high)	1(0.44–2.35)	0.94	**3.96 (1.2–12.7)**	0.02*
**RAB7** (low vs high)	1.62(0.73–3.6)	0.22	0.39 (0.11–1.3)	0.12
**SPINK1** (low vs high)	1.9 (0.8–4.3)	0.13	0.69 (0.18–2.56)	0.58

*p ≤ 0.05 according to Cox test; HR: hazard ratio; CI: confidence interval.

**Figure 5:**
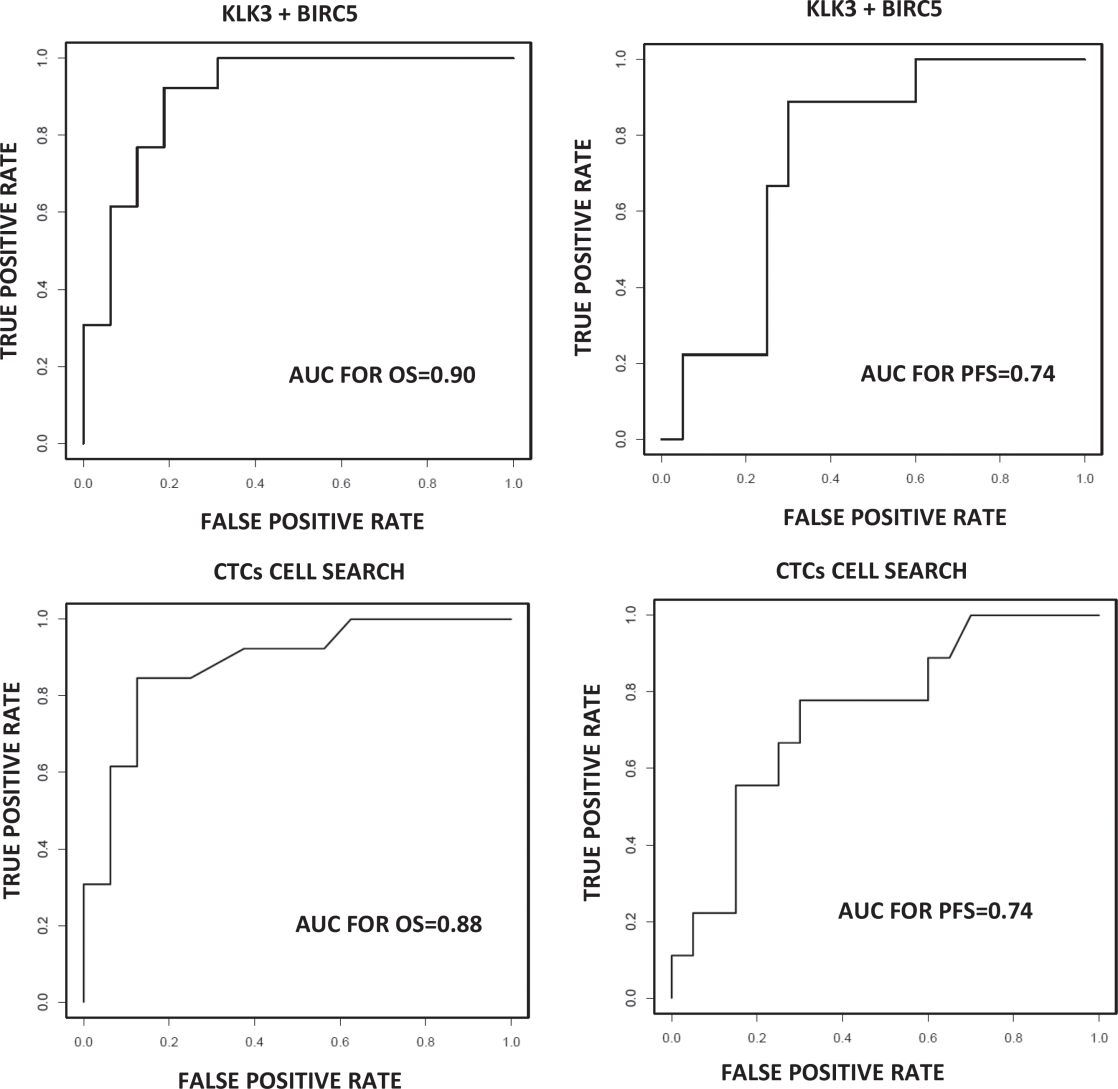
Prognosis value for a logistic model combining KLK3 and BIRC5 compared with CellSearch system ROC-curve showing the sensitivity and specificity of CellSearch and the combination of KLK3 and BIRC5 to detect the presence of CTC in our mCRPC cohort.

Finally, we generated a logistic model with the panel of CTC-biomarkers in order to determine which marker combination has the highest diagnostic and prognostic value. For this, linear regression was realized using the numeric values for each biomarker. We found the best performance with an AUROC value of 0.74 and 0.9 for PFS and OS respectively using the combination of *KLK3* and *BIRC5* (Figure 5). Importantly the AUROC for OS is higher that showed by CTCs count after CellSearch analysis at baseline (Figure 5).

Overall, these results demonstrated the clinical value of CTCs count and the marker panel identified in the CTC population of our cohort of mCRPC patients, providing an easy method to determine the group of patients that will show a better response to the chemotherapy treatment after the androgen deprivation therapy.

## DISCUSSION

Nowadays it is well accepted that CTCs provide a uniquely accessible source of tumor-derived material for molecular analyses, even more important in tumors such as PCa where the inaccessible metastatic lesions not allow individualize therapies according to the mechanism of drug resistance, which appear during the evolution of the disease [25, 26]. Previous studies have shown that both baseline CTC count and CTC changes during chemotherapy or hormone- based treatments in mCRPC patients were more closely associated with patient survival than were PSA changes [5]. With these results, CellSearch system obtained the FDA approval for CTC counting in patients with mPCa.

The results in our cohort of patients are in accordance with those described previously using CellSearch and confirm the value of CTC monitoring during the treatment with chemotherapy in mCRPC patients [7, 12, 27]. We found that the presence of ≥ 5 CTCs/7.5 mL at 3^rd^ and 6^th^ cycle was associated with a very high risk of being in biochemical or radiological progression. This fact, together with the higher PFS and OS showed in patients who changed from ≥ 5 CTCs/7.5 mL to < 5 CTCs/7.5 mL after the 6^th^ of chemotherapy reinforce the value of CTCs enumeration to manage mCRPC patients.

In addition to the CTC enumeration, the molecular characterization of CTCs could provide important insights into disease progression and evolution. Our group and others previously demonstrated that combination of CTC-immunoenrichment and the analysis of CTC-transcriptome by RT-qPCR provide an alternative and high sensitivity method for CTCs detection and characterization [21–23, 28]. Here we use this approach to characterize the CTC population from mCRPC (patients progressing despite castrate levels of testosterone and after at least one hormonal manipulation) that will start systemic chemotherapy based on Docetaxel/Cabazitaxel in order to identify CTC-markers with clinical impact for the management of these patients.

We found a CTCs transcriptome phenotype mainly characterized by the expression of two groups of genes; those related with androgen signaling pathway such as *AR* and *CYP19* and those implicated in relevant functions for PCa progression and resistance to chemotherapy such as *BIRC5*, *TUB1A*, *GDF15*, *RAB7* and *SPINK1*.

The analysis of AR in CTCs was attempted previously by various groups with promising results [29]. For example recent studies proposed the evaluation of AR modifications in CTCs, including detection of AR-V7 and point mutations, as an accessible and valuable tool for treatment selection [30–32]. Besides AR alterations, the overexpression of enzymes responsible for androgen synthesis and metabolism has been also proposed to explain the persistence of hormone-mediating signaling in prostate tumor cells under hormone deprivation state [33]. In this sense we found that CYP19A1 was present in the CTC population of our mCRPC patients suggesting that patients progressing after androgen deprivation could present adaptative mechanisms to maintain the hormone stimulation of prostate tumor cells.

On the other hand, we identified *BIRC5, TUB1A, GDF15, RAB7* and *SPINK1* as genes characterizing CTCs of mCRPC patiens. They conform a diverse group of genes with a common role promoting tumor aggressiveness and the development of resistance to taxanes-based treatment [34–37]. For example, BIRC5 (survivin) expression in PCa tissues has been related with high Gleason score, chemoresistance and cancer progression [35]. In addition SPINK1 overexpression has also been associated with an increased risk of biochemical recurrence in hormonally and surgically treated prostate cancer cohorts [36] while enhanced level of GDF-15 in prostate tumor cells has been also associated with their acquisition of epithelial-mesenchymal transition phenotype and docetaxel resistance, even in PCa stem/progenitor cells [37]. It seems that the expression of these genes could provide CTCs with mechanisms to resist the therapy pressure.

Importantly, in addition to provide more information about the biology of the specific subpopulation of CTCs in mCRPC, our gene-expression profiling also permitted the identification of valuable prognostic biomarkers. Thus, high levels of AR, CYP19 and GDF15 were associated with poor PFS rates while AR, GDF15 and BIRC5 were also found as consistent predictors of OS. Importantly, in our cohort of patients CTCs-PSA levels at baseline showed more clinical relevance than serum PSA levels in terms of both PFS and OS. Reinforcing the value of our study, the combination of KLK3 and BIRC5 expression showed higher value than CellSearch to discriminate patients with a poorer outcome. Thus, our molecular CTCs-signature could be of great value to personalize the treatment in mCRPC providing a potential tool to monitor therapy or predict the clinical response because all the genes are implicated in critical functions to modulate anti-tumor therapies activity. However, further analysis in a large multicenter study including therapy monitoring should be done to determine the clinical value of these markers.

In conclusion the present study provides with a promising and useful alternative method in mCRPC for: a) CTCs monitoring during the chemotherapy administration; b) anticipating the biochemical and radiological progression; c) the dynamic characterization of CTCs focusing on the expression of genes associated to taxanes resistance.

## MATERIALS AND METHODS

### Patients

A total of 29 mCRPC patients and 19 healthy individuals were prospectively enrolled at Complexo Hospitalario Universitario de Santiago, Santiago de Compostela (Spain), from 2011 to early 2014. Participants were informed and signed consent was given before their inclusion in the study according to the Galician Ethical Committee. All individuals in the PCa group had histological confirmed diagnostic of adenocarcinoma, evidence of progression despite castrate levels of testosterone and at least one hormonal manipulation failed, being eligible for systemic chemotherapy based on Docetaxel/Cabazitaxel. Other inclusion criteria were an Eastern Cooperative Oncology Group (ECOG) performance status not greater than 2 and an estimated OS higher than 3 months. Detailed information about patients included in the analysis is available in Table 5. Control group included 19 healthy volunteers with similar ages range and no previous cancer episodes.

**Table 5:** Demographics of patients included in the study

	*n* (%)
**ECOG**	
0	7 (24,1)
1–2	22 (75,9)
**Gleason score at diagnosis**	
≤ 7	15 (51,7)
> 7	11 (37,9)
unknown	3 (10,4)
**Lymph nodes metastasis**	
no	17 (58,6)
yes	12 (41,4)
**Metastasis site**	
Bone	29 (100)
Visceral	4 (13,8)
**Number of prior treatments regimens**	
1–2	15 (51,7)
> 2	14 (48,3)
**PSA at baseline** (ng/dl)	121 (12–3238)*
Lactate Deshydrogenase baseline (U/l)	454 (121–1136)*
**Alkaline phosphatase baseline** (U/l)	320 (77–3115)*

*median (min-max).

### CTC isolation and molecular characterization

Two parallel methods were used to isolate and analyze CTC population. For CTCs enumeration 7,5 mL of peripheral blood (CellSave, Veridex LLC) were obtained and maintained at room temperature (within 96 h of collection) until their analysis using CellSearch System (Janssen Diagnostics). Cells expressing EpCAM were immunomagnetically enriched from 7.5 mL of blood and fluorescently labelled with DAPI, CD45-APC, and CK-PE. Then the images of stained cells were acquired by a semiautomatic fluorescence microscopy system. Finally, two experimented reviewers selected CTCs following CellSearch guideline from the gallery of objects proposed by the system [4]. Thus, we selected as CTCs those cells with nearly round or oval morphology, total cell size of at least 4 μm, cytokeratin (8, 18, and/or 19 ) positive staining while CD45 negative staining and nucleated (DAPI positive staining).

For CTC molecular analysis we combined an EpCAM-based CTC inmunoisolation and a RT-qPCR analysis for a panel of genes [21]. In brief, CTC isolation was made according to manufacturer´s instructions with CELLection^TM^ Epithelial Enrich system (Invitrogen, Dynal, Oslo, Norway) that contains beads coated with EpCAM antibodies. Total RNA from CTC was extracted with the QIAmp viral RNA mini kit (Qiagen, Valencia, CA, USA), designed for very low cellularity samples. cDNA was synthesized by using Superscript III reverse transcriptase (Invitrogen) and subjected to a preamplification for 14 cycles with TaqMan^®^ PreAmp Master Mix kit (Applied Biosystem, Foster City, CA, USA) prior to RT-qPCR (TaqMan Gene Expression Assays; Applied Biosystems), to maximize detection rates.

The assay included the following 15 relevant genes for PCa progression: *AR, CYP19* and *CYP17* because they are genes implicated in androgen-pathway regulation that plays a critical role in prostate cells biology [38, 39]; *CD133, CD44, ALDH1A*, *ABCG2* and *CD49f* as genes related to stem cell phenotype associated to intrinsic therapeutic resistance against androgen-blockage and chemotherapy [40, 41] and previously described as relevant for CTCs molecular features [42]; and *BIRC5, CLU, GDF15, RAB7A, SPINK1, TUB1A, MDR1* that are genes implicated in PCa aggressiveness and/or resistance to taxanes [34–37, 43–45] and, therefore, highly interesting markers to be analyzed in the CTCs population from mCRPC patients. The house-keeping gene GAPDH as total cellular load marker was analyzed to detect the presence of an additional cell population in the blood of patients as described previously [23, 46] and demonstrated in the present study by spiking experiments using PC3 cells (see Supplementary Figure 1). Probe characteristics are detailed in Supplementary Table 1. Data were analyzed using StepOne Software v.2.1. (Applied Biosystems). Mean threshold cycle (depicted as 40-Ct) for every candidate gene was normalized to CD45 that allows the quantification of non-specific isolation and that have been previously used to discriminate the population of CTCs from the total of nucleated cells isolated after the immunoenrichment step [23, 46, 47]. Samples were run in duplicate and all plates included negative controls. The protocol was also applied to healthy volunteer's blood for subtracting the unspecific background.

We analyzed the expression levels of these genes in the whole set of patients and controls and identified those genes with a significant expression in CTC from the group of patients compared to the background of unspecific isolation from the controls.

### Statistical analysis

Data were analysed using SPSS (Chicago, version 15.00 for Windows) and GraphPad Prism 4.00 software (GraphPad Softwares Inc, San Diego, CA, USA). Differences of gene expression between patients and controls were analyzed Using Mann-Whitney test. PFS and OS were analyzed using Kaplan-Meier analysis and differences were examined by log-rank test. Univariate and multivariate analyses were performed using Cox regression statistics. Bivariate correlation analysis was routinely carried out according Pearson statistic while Ʃ^2^ test was used for correlations between categorical variables. *p* values < 0.05 were considered statistically significant.

## SUPPLEMENTARY MATERIALS FIGURE AND TABLES



## Abbreviations

ABCB1 (MDR1): ATP binding cassette subfamily B member 1
ABCG2: ATP binding cassette subfamily G member 2
ALDH1A1: aldehyde daehydrogenase 1 family member A1
AR: androgen receptor
BIRC5: baculoviral IAP repeat containing 5
CD133: CD133 antigen or prominin1
CD44: CD44 antigen
CD49F: CD49 antigen-like family member F
CLU: clusterin
CTCs: circulating tumour cells
CYP17A1: cytochrome P450 family 17 subfamily A member 1
CYP19A1: cytochrome P450 family 19 subfamily A member 1
ECOG: Eastern Cooperative Oncology Group
GDF15: growth differentiation factor 15
KLK3/PSA: kallikrein related peptidase 3/prostate specific antigen
LDH: lactate dehydrogenase
mCRPC: metastasic castration resistant prostate cancer
OS: overall survival
PA: phosphatase alkaline
PCa: prostate cancer
PFS: progression free survival
PSA: prostate specific antigen
RAB7A: ras-related protein Rab-7a
SPINK1: serine peptidase Inhibitor, Kazal Type 1
TUB1A1: tubulin alpha 1a
