# Supplementary material for: Improving circulating tumor cells enumeration and characterization to predict outcome in first line chemotherapy mCRPC patients
Source: Oncotarget. 2017 May 19;8(33):54708–21. doi: 10.18632/oncotarget.18025 (PMC5589615; doi:10.18632/oncotarget.18025)
Supplement: Supplementary file 1 [file oncotarget-08-54708-s001.pdf]

Improving circulating tumor cells enumeration and characterization to predict outcome in first line chemotherapy mCRPC patients

Supplementary Materials

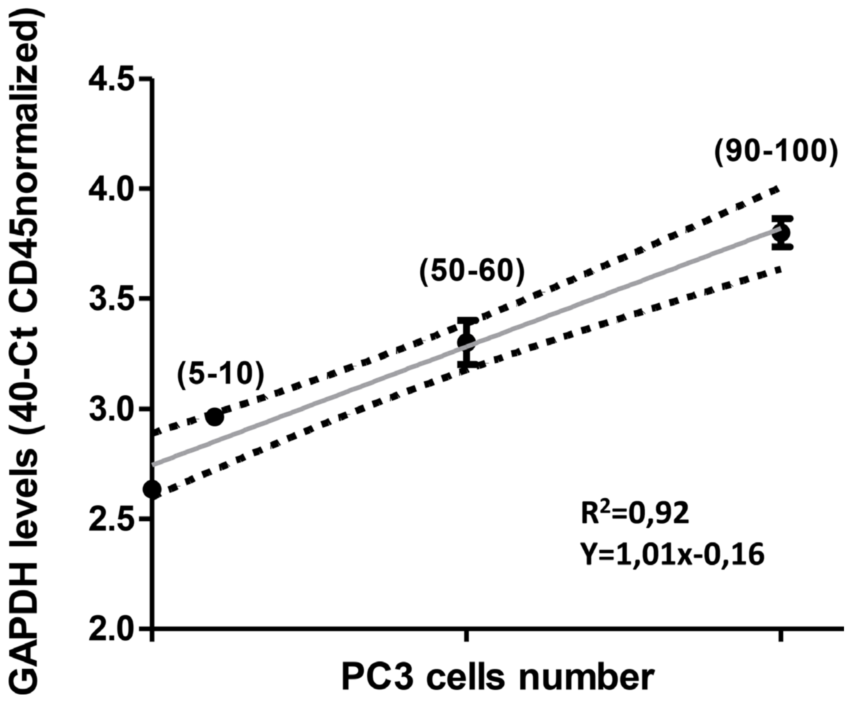

Supporting Figure 1: GAPDH expression levels after immunoisolation of PC3 cells from blood of healthy donors. Model CTC samples were prepared by spiking PC3 cells (cultured in DMEM culture medium) into 7,5 mL of healthy donor blood in concentrations ranging from 5–10, 50–60, and 90–100 per tube in two independent experiments. Immunoisolation, RNA extraction, cDNA synthesis, pre-amplification and RT-qPCR were performed as described in Materials and Methods section. GAPDH levels (40-Ct) were normalized with CD45 (40-Ct) to substract the background of blood cells unspecifically isolated. We were able to detect the precence of PC3 cells at a range of 5-10 cells/7,5 mL of blood. R^2 value was 0,92.

Supplementary Table 1: TaqMan RT-qPCR probes used for the gene expression analysis

Gene	TaqMan assay	Gene	TaqMan assay
GAPDH	Hs99999905_m1	BIRC5	Hs04194392_s1
PTPRC (CD45)	Hs00894734_m1	CLU	Hs00156548_m1
CD133	Hs01009250_m1	GDF15	Hs00171132_m1
ALDH1A1	Hs00946916_m1	RAB7A	Hs01115139_m1
CD49F	Hs01041011_m1	SPINK1	Hs00162154_m1
AR	Hs00171172_m1	TUB1A1	Hs00362387_m1
KLK3	Hs02576345_m1	MDR1 (ABCB1)	Hs00184500_m1
CD44	Hs01075861_m1	CYP17A1	Hs01124136_m1
ABCG2	Hs01053790_m1	CYP19A1	Hs00903413_m1

Supplementary Table 2: Association between CTCs count by cellSearch and clinico-pathological characteristics

Characteristics	CTCs levels < 5		CTCs levels ≥ 5		*p*
Locally advanced disease at diagnosis	*n* = 3	20 %	*n* = 12	80 %	0.23
Nodal invasion (cN)	*n* = 2	22.3%	*n* = 7	77.7%	0.62
Gleason score > 7	*n* = 3	27.2%	*n* = 8	72.8%	0.68
Visceral disease	*n* = 1	20%	*n* = 4	80%	0.63
Baseline PA, mean	*n* = 10	207	*n* = 18	765	0.02*
Baseline LDH, mean	*n* = 7	396	*n* = 17	558	0.04*
Baseline PSA, mean	*n* = 10	107	*n* = 19	580	0.08

$p \leq 0.05$ according to X^2 or *non-paired *T* test.

Supplementary Table 3: Correlation between baseline characteristics and CTCs profile

	KLK3 (mean)	AR mean)	TUB1 (mean)	CYP19 (mean)	BIRC5 (mean)	RAB7 (mean)	SPINK1 (mean)	GDF15 (mean)
**ECOG**								
0 (*n* = 7)	–7.69	**–7.16***	3.28	–11.44	1.53	3.59	–16.55*	–13.43*
1–2 (*n* = 22)	–5.17	**–3.49***	1.75	–9.42	1.98	2.93	–11.92*	–6.85*
**Gleason score at diagnosis**								
≤ 7 (*n* = 15)	–3.86	–5.33	**1.24***	–9.80	1.44	2.39	–12.37	–8.78
> 7 (*n* = 11)	–5.53	–6.23	**3.34***	–10.09	2.60	3.98	–13.91	–9.10
**Lymph nodes metas.**								
No (*n* = 17)	–4.69	–4.84	2.43	–10.29	1.59	2.96	–13.06	–9.23
Yes (*n* = 12)	–6.93	–3.73	1.68	–9.36	2.27	3.27	–13.01	–7.32
**Number of prior treatments regimens**								
1–2 (*n* = 15)	–4.69	–3.14	2.23	**–8.78***	2.52	3.57	–11.74	**–5.97***
> 2 (*n* = 14)	–6.93	–5.70	2.01	**–11.12***	1.18	2.58	–14.43	**–11.08***
**PSA at baseline**								
< 122 (*n* = 15)	–6.23**	–4.92	2.02	–10.07	1.60	2.87	–12.95	–9.41
≥ 122 (*n* = 14)	–4.33**	–2.66	2.44	–9.40	2.72	3.80	–13.32	–5.38
**LDH baseline**								
< 454 (*n* = 12)	–6.40	–5.03	1.76	–9.44	1.62	3.21	–12.78	**–9.12****
454 (*n* = 12)	–4.49	–2.85	2.44	–9.60	2.64	3.21	–11.80	**–6.08****
**PA baseline**								
< 320 (*n* = 14)	–7.28	**–5.62****	2.09	–9.98	1.80	3.27	–13.68	**–11.20****
≥ 320 (*n* = 14)	–4.43	**–3.24****	2.10	–10.05	1.97	2.94	–12.09	**–5.58****

*$P \leq 0.05$ according to *T*-test; **$p \leq 0.05$ according to Pearson test.

Supplementary Table 4: Cutoff values used to define the high/low group for each CTCs-marker based on 50, 60 and 70 percentiles

Gene	Percentile	Cutoff value
KLK3	P50	–3.8
AR	P50	–3.8
CYP19	P70	–7.5
TUB1	P70	3.5
GDF15	P70	–6.11
BIRC5	P70	2.8
RAB7	P50	3.1
SPINK1	P70	–11.6
